# Application of bilateral simultaneous sequential single-incision video-assisted thoracic surgery in multiple nodules both lungs: a single-center experience of 10 cases

**DOI:** 10.1186/s12893-022-01841-3

**Published:** 2022-11-10

**Authors:** Wensong Shi, Yuzhui Hu, Guotao Chang, Huiyu Zheng, Zhiqiang Yang, Xiaogang Zhao, Yulun Yang, Xiangnan Li

**Affiliations:** 1 Department of Thoracic Surgery, The First Affiliated Hospital of Zhengzhou University, Zhengzhou, 450052 China; 2 Department of Thoracic Surgery, The Fifth Clinical Medical College of Henan University of Chinese Medicine/Zhengzhou People’s Hospital, Zhengzhou, 450052 China; 3 Department of Geratology, Ninth People’s Hospital of Zhengzhou, Zhengzhou, 450053 China; 4 Department of Thoracic Surgery, Shanghai Pulmonary Hospital Affiliated to Tongji University, Shanghai, 200433 China

**Keywords:** Multiple nodules in both lungs, Single-incision video-assisted thoracic surgery (U-VATS), Bilateral simultaneous sequential thoracic surgery, ERAS

## Abstract

**Objective:**

To discuss the application of bilateral simultaneous sequential single-incision video-assisted thoracic surgery in multiple nodules in both lungs.

**Methods:**

A retrospective analysis of 10 patients in Zhengzhou People’s Hospital who underwent single-incision thoracoscopic surgery to treat multiple nodules in both lungs at the same time from September 2019 to January 2021, and analyze the perioperative indicators (general condition, smoking history, family history, follow-up time of pulmonary nodules, size, location, height and weight, pulmonary function, intraoperative blood loss, operation time, color and volume of drainage fluid, catheterization time, perioperative complications, length of stay, pathology, patient satisfaction, etc.).

**Results:**

All 10 patients used single-incision thoracoscopy to complete bilateral simultaneous sequential operations, aged 32 to 70 years, 8 female patients, 2 male patients, preoperative follow-up time ranging from 1 day to 2 years, a total of 23 lung nodules were removed except for the benign lesions in one nodule in the 2 patients, the other nodules were tumorous lesions (91.3%). The average total hospital stay was 10.5 days (8–14 days), and the average operation time was 194.5 min (145–292 min). The blood loss ranged from 10 to 280 ml, all patients had no serious complications during the perioperative period, and they recovered well and were discharged smoothly, and the satisfaction reached 100%.

**Conclusion:**

Single-incision bilateral simultaneous sequential thoracoscopy have certain advantages in the treatment of patients with multiple nodules in both lungs, conforms to the concept of rapid recovery, and is a feasible choice in the shared decision making of doctors and patients.

## Background

In 2011, the US-based National Lung Screening Trial (NLST) found that annual screening with low-dose computed tomography (LDCT) substantially reduced mortality by 20% relative to conventional chest x-ray screening from lung cancer [1]. With public awareness comes early detection, early diagnosis and early treatment are of key importance in preventing disease development, and LDCT is widely used for evaluation of lung disease, the detection rate for pulmonary nodules has been significantly improved. Pulmonary nodules are approximately round lesions that are defined as less than 3 cm in diameter and that are completely surrounded by lung parenchyma, without other abnormalities, and were classified as solitary or multiple lung nodules, depending on the number. Multiple lung nodules presenting as multifocal ground-glass nodules (multi-GGN) on computed tomography scan, and should be staged as multiple primaries instead of intrapulmonary metastases, they often indicate multiple primary lung cancer and pathologically adenocarcinoma. The lesions with the largest diameter among multiple nodules are called primary lesions, and the rest are called secondary lesions. The management of multiple pulmonary nodules is still debated. With increasing experience, the indications for single-incision video-assisted thoracic surgery have been expanded, and were also employed in the bilateral simultaneous thoracic surgery, our team has some experience in this field recent years, and the report is as follows.

## Materials and methods

We retrospectively reviewed the medical charts of 10 patients with bilateral pulmonary nodules who underwent bilateral simultaneous single-incision VATS in the Department of Thoracic Surgery of Zhengzhou People’s Hospital from September 2019 to January 2021. Perioperative related indicators were collected, including the general information of patients, smoking history, family history, height and weight, preoperative pulmonary function; follow-up time, size and location of pulmonary nodules; intraoperative rapid freezing pathology results, intraoperative blood loss, operation time; color and volume of drainage fluid, catheterization time, perioperative complications, total hospital stay, postoperative pathology, patient satisfaction, etc.

Inclusion criteria: Bilateral multiple lung nodules with a size < 3 cm and were considered tumor nodules after multidisciplinary team consultation; This should have been an indication for surgical excision of limited pneumonectomy (total tumor size ≤ 3 cm and consolidation tumor ratio ≤ 0.5 [2–4]); The patient tolerated the procedure well and without apparent abnormal cardiac or pulmonary function during the perioperative examination.

Exclusion criteria: With sufficient preoperative evaluation, those patients who were unsuitable for bilateral simultaneous surgery or refused this surgical plan; Patients who were expected to have a total resection of more than 8 lung segments; A history of hemopneumothorax and pleurisy; Those with severe underlying diseases that have not been controlled and stabilized.

### Surgical procedure

The proper preoperative planning and pulmonary resection procedures were developed and implemented after all patients received preoperative evaluation to assess their physical conditions, based on the size, location, and characteristics of nodules, as well as the performance status and pulmonary function testing. And under the physician–patient shared decision-making as well. Pulmonary nodules were localized by CT-guided hook-wire in all patients. Simultaneous bilateral pulmonary resection by U-VATS was performed by the same team of surgeons and anesthetists using double-lumen endotracheal intubation. The trachea was cannulated in the side of less resection of lung tissue anticipated to prevent affecting the safety of surgery. Lateral position, the side with more lung tissue anticipated to be resected side down. The 4th or 5th intercostal space between the anterior axillary and the axillary middle line as surgical incision of about 3 cm was used as the endoscopic and operative holes. Frozen-section pathological examinations were performed on all resected specimens intraoperatively. And the wedge resection, pulmonary segment resection or pulmonary lobectomy was depended on it. Two drainage tubes was routinely placed after the operation, a thick drainage tube (20F) was placed at the top of the chest through the dorsal side of the incision, and another thin one (10F) like central venous catheter was placed percutaneously through the 7th intercostal space on the posterior axillary line. Air and bloody liquid was evacuated via the drainage tube when we perform another side operation. A U-shaped cushion was positioned below the body used to maintain an unobstructed drainage tube. The same procedure was performed on the contralateral side.

### Follow-up

All patients were followed up with chest CT and serum tumor markers [carcinoembryonic antigen (CEA), cytokeratin 19 fragment (CYFRA 21-1), squamous carcinoma-associated antigen (SCC), neuron specific enolase (NSE)] every 3 months and with cranial MRI and abdominal CT (including adrenals) every half year, and whenever necessary with positive symptoms at our outpatient clinic.

## Results

All 10 patients underwent simultaneous bilateral pulmonary resection by single-incision VATS. Our patients’ cohort comprised 2 men and 8 women, with mean age 52.8 (range 28–70) years. Preoperative follow-up time for pulmonary nodules from 1 day to 2 years. Pieces of lungs were removed, and the nodules were counted. A total of 23 lung nodules were resected, the nodules except for the benign lesions on one side of the nodules in 2 patients were tumor lesions (91.3%). The average total hospital stay was 10.5 (range 8–14) days, the average operation time was 194.5 (range 145–292) min, and the blood loss was 10–280 ml. We encountered no major perioperative complications in this group, the patient recovered well from surgery and discharged from our department uneventful with a satisfaction level of 100%. The details are showing in Table 1.

**Table 1:** Detailed analysis of 10 patients with simultaneous bilateral pulmonary resection by single-incision VATS

Sex	Age (year)	Resection (right/left)	Preoperative follow-up time	FEV1/%	MVV/%	Pathological examinations of the right lung	Pathological examinations of the left lung	Long of stay/d	Operation time/min	Bleeding/ml
Female	32	RLo/LW	10 m	93	102	MIA	AAH	10	145	50
Female	63	RW/LW	21 d	90.2	86.1	BN	AIS	13	205	50
Female	60	RW/RW/LW	7 m	94	98	AIS AAH AAH	AAH	10	180	50
Female	44	RW/LW	1 d	89.9	92	MIA AIS	BN	8	205	10
Female	55	RW/LW	2 m	80	80	MIA	AIS	9	150	10
Male	56	RW/LS	4 m	80	93	AAH	MIA	11	230	280
Male	52	RW/LW	3 m	87	95	MIA	IAC	10	220	50
Female	68	RW/LW	3 m	84	75	MIA	MIA	9	150	50
Female	70	RW/LLo	1 y	77	92.6	MIA	IAC	14	292	100
Female	28	RW/LW	2 y	97.5	82.2	MIA	MIA	11	172	100

*R* right, *L* left, *Lo* lobectomy, *S* segmentectomy, *W* wedge resection, *AAH* atypical adenomatous hyperplasia, *AIS* adenocarcinoma in situ, *MIA* minimally invasive adenocarcinoma, *BN* benign nodules, d day, m month, y year

## Discussion

The detection rate for pulmonary nodules, particularly multiple pulmonary nodules, has been significantly improved. Follow-up and observation, bilateral procedures, staged surgeries, surgery combined with radiofrequency ablation, irradiation are options, each have their own indications and advantages and disadvantages. Treatment regime should be individualized for patients. There were lots of confusion they were facing among the diagnose and treatment of the nodules. Clinicians often need to make a decision after careful evaluation in terms of patient willingness, economics, technical feasibility, and safety.

For patients with bilateral disease we intend to do simultaneous surgery, we used to perform median thoracotomy, bilateral intercostal incision with vertical midline extension, Mercedes-type, or bilateral posterolateral incision. It gradually decreases with time in all indications because the more traumatized and cardiorespiratory function affected except bilateral lung transplantation. There are certain strengths of bilateral simultaneous surgery: the cost, length of total hospital stay, risk presented the secondary anaesthetic and surgery, tremendous psychological pressure on patients and their relatives, risk of tumor progression and in-hospital complications have decreased, and return a patient to normal life as quickly as possible [5–9]. With the increasing use of laparoscopic and thoracoscopic surgery, particularly the expand indications of single-incision VATS [10, 11], patients undergoing fast-track rehabilitation suffer from less pain and have a faster return in the postoperative course. It’s possible for patients with bilateral disease who underwent bilateral operation at one operative setting.

Surgeons have been fervent about this innovative method since as early as the 1950s [8, 12, 13]. Lunxu Liu, from the Department of Thoracic Surgery of West China Hospital of Sichuan University, demonstrates the value of single-port thoracoscopic surgery in treating multiple primary lung cancers in 2014. Further exploration of this method after this, the majority of the lesions involved bilateral lungs disease like bilateral isolated metastases [14], bilateral pulmonary bullae with unilateral/bilateral pneumothorax and lung volume reduction surgery for patients with severe emphysema [15]. And may involve empyema [16], bilateral pleural biopsies, bilateral thoracic sympathectomy [17, 18] and thymoma complicated with myasthenia gravis, etc.

With the advances in surgical and anesthetic techniques, especially the double-lumen endotracheal tube (DLT) technology progresses, and it’s essential for thoracic surgery that requires one-lung ventilation, those all have played a positive role in development of the field of thoracic surgery [18]. Bilateral simultaneous surgery has greater impact on ventilation/blood flow, and is a challenge in anesthesia research as well. Surgeon usually as a staged procedure with the section of less lung tissue side done first, it is better for the attending anesthesiologists to apply the airway plan for tracheal intubation with a DLT and isolating each lung with the main airway on the side with more remaining lungs. During surgery, single contralateral lung ventilation was used, and the other lung were mechanically ventilated to maintain normocapnia. Maintaining the unobstructed drainage tube when the same procedure was repeated on the other side is another key factor. In operatively, patients were treated with ventilator-assisted ventilation to achieve peripheral oxygen saturations above 90%.

Patient with worse pain may be more likely to have bilateral surgery than unilateral surgery. After the operation, the intravenous pump is routinely placed and non-steroidal analgesics were used for active and appropriate pain management in our department. In addition, we use double-tube drainage to drain the effusion, a thick thoracic drainage tube was routinely indwelled in the surgical incision, and a thin thoracic drainage tube was indwelled in the seventh intercostal space of the midaxillary line below the incision. When the drainage volume in the surgical area was no obvious bubble formation, the colour of the drainage fluid became clear and the thin tube was unobstructed, the thick drainage tube was removed as possible and alleviate the pain to help them rehabilitate as soon as possible.

Nursing staff requirements highly for bilateral simultaneous surgery, high-quality care which used during perioperative period are helpful to the recovery of patients. At the same time, a correct understanding of superior and inferior of bilateral simultaneous operation were given preoperatively to the patients, was helpful to avoid overtreatment and alleviate the family’s anxiety. Effects of timing and reinforcement of preoperative education on illness education, disease management and recovery of patients having surgery.

The application of rapid recovery protocols may accelerate the recovery of these patients, reduce the hospital stay and recognized by the wide medical community and patients. Bilateral sequential operation conformed to the rapid rehabilitation surgery concept and may be suitable and beneficial in clinical practice and helpful to the recovery of patients. There is currently no standard guideline and large clinic-based studies when selecting patients for bilateral surgery. The rationale for selection was based on surgical operator’ own clinical experience. After we managed the journals searches [2–7, 9, 14, 15, 17–24] and summaries of related work experience in our department, there are several caveats to our study that are noted throughout and summarized below.

① Such patients without a complicating pleural infection, thoracic trauma with hemopneumothorax and rib fractures, a history of prior pleurodesis or any subtle pleural nodularity or thickening is found on preoperative chest CT scans;
② With appropriate monitoring of cardiopulmonary parameters it can be performed safely and is well tolerated. Walking up 3-story fall without resting at their normal pace. Pulmonary function FEV1 > 80% and MVV > 70%, the range is less than or equal to 8 segments based on the anatomy of the bronchial tree expected, the ability to tolerate single-lung ventilation;
③ With the lung nodule preoperative localization by Hookwire or other methods;
④ Except for the right middle lobe combined with left lung limited pneumonectomy, it is not recommended to combine the other lung lobes with contralateral pneumonectomy, and at least one side should be subjected to limited pneumonectomy;
⑤ When attending anesthesiologists perform double-lumen tracheal intubation, it is recommended that the side with the expected less lung resection be the main tube;
⑥ Soft and thin drainage tubes and combined drainage of upper thick tube and lower thin tube were recommend for bilateral thoracic drainage tubes, it is helpful to remove the thick tube as soon as possible after operation for quick recovery after operation;
⑦ The intravenous pump combine non-steroidal analgesics were used for adequate analgesia, and coughing and sputum were recommended for protection against respiratory infection;
⑧ Pay attention to the infusion volume and rate to alleviate the lung edema and injury and prevented cardiac insufficiency;
⑨ The patient should be placed in a semirecumbent position and was encouraged to be out of bed the first postoperative day as soon as possible, for pleural fluid obey gravity and favor a net movement.

We acknowledge that it has some limitations, the number of cases in this group is small and most of them are limited pneumonectomy. In clinical application, it is necessary to fully consider the comprehensive condition of the patients and individualized treatment plans need to be formulated to improve the adherence of patients.

## Conclusions

In summary, the difficulty is how to choose bilateral surgery simultaneous and staged bilateral surgery in multiple nodules both lungs. Simultaneous bilateral pulmonary resections by single-incision VATS is a feasible and safe procedure in appropriate patients. However, it should be noted that “being proficient before surgery, cautious during surgery, and diligent after surgery” facilitates rapid recovery of patients.

With the range operative indications for subxiphoid surgical window, it avoids the change of position during the bilateral operation and increases the duration of surgery and anesthesia to some extent, also has some advantages [15, 21], and we hope their superior and inferior will be confirmed by a larger study. The treatment by a combination of ablation procedure ablative therapy, stereotactic radiotherapy and surgery [23, 25] may minimize the risk of surgical adverse events. It’s of special value for patients who are unable to tolerate bilateral surgery.

## Data Availability

All data generated or analysed during this study are included in this published article.

## Abbreviations

VATS: Video-assisted thoracic surgery
NLST: National Lung Screening Trial
LDCT: Low-dose computed tomography
CEA: Carcinoembryonic antigen
CYFRA 21-1: Cytokeratin 19 fragment
SCC: Squamous cancinoma-associated antigen
NSE: Neuronspecific enolase
DLT: Double-lumen endotracheal tube
R: Right
L: Left
Lo: Lobectomy
S: Segmentectomy
W: Wedge resection
AAH: Atypical adenomatous hyperplasia
AIS: Adenocarcinoma in situ
MIA: Minimally invasive adenocarcinoma
BN: Benign nodules
d: Day
m: Month
y: Year

## References

[1] Aberle DR, Adams AM, Berg CD (2011). Reduced lung-cancer mortality with low-dose computed tomographic screening. N Engl J Med.

[2] Ito H, Suzuki K, Mizutani T (2020). Long-term survival outcome after lobectomy in patients with clinical T1 N0 lung cancer. J Thorac Cardiovasc Surg.

[3] Suzuki K, Saji H, Aokage K (2019). Comparison of pulmonary segmentectomy and lobectomy: safety results of a randomized trial. J Thorac Cardiovasc Surg.

[4] Sun K, You A, Wang B (2021). Clinical T1aN0M0 lung cancer: differences in clinicopathological patterns and oncological outcomes based on the findings on high-resolution computed tomography. Eur Radiol.

[5] Zheng H, Peng Q, Xie D (2021). Simultaneous bilateral thoracoscopic lobectomy for synchronous bilateral multiple primary lung cancer-single center experience. J Thorac Dis.

[6] Lin S, Yang C, Guo X (2021). Simultaneous uniportal video-assisted thoracic surgery of bilateral pulmonary nodules. J Cardiothorac Surg.

[7] Huang C, Sun Y, Wu Q (2021). Simultaneous bilateral pulmonary resection via single-utility port VATS for multiple pulmonary nodules: a single-center experience of 16 cases. Thorac Cancer.

[8] Yim AP (1996). Simultaneous vs staged bilateral video-assisted thoracoscopic surgery. Surg Endosc.

[9] Qu R, Hao Z, Zhang Y, Bie L, Fu X, Zhang N (2020). Single-center experience of simultaneous bilateral uni-portal video-assisted thoracoscopic surgery for multiple ground-glass opacities. J Cardiothorac Surg.

[10] Migliore M, Halezeroglu S, Molins L (2016). Uniportal video-assisted thoracic surgery or single-incision video-assisted thoracic surgery for lung resection: clarifying definitions. Future Oncol.

[11] Halezeroğlu S (2018). Single incision video-assisted thoracic surgery pneumonectomy for centrally located lung cancer. Future Oncol.

[12] Merlier M, Leguerrier A, Bouquet P, N'Guimbous JF (1977). Surgery of 19 bilateral simultaneous pulmonary metastases. J Chir (Paris).

[13] Trigo ER, Villegas AH, Paz MR, Roncoroni AJ (1957). Massive bilateral bullous emphysema; simultaneous bilateral surgery & functional data; case report. Prensa Med Argent.

[14] Feldman HA, Zhou N, Antonoff MB (2021). Simultaneous versus staged resections for bilateral pulmonary metastases. J Surg Oncol.

[15] Li X, Wang X, Zhang H, Cheng H, Cao Q (2019). Unilateral single-port thoracoscopic surgery for bilateral pneumothorax or pulmonary bullae. J Cardiothorac Surg.

[16] Nose N, Anami T (2014). Simultaneous bilateral decortications via video-assisted thoracic surgery for bilateral empyema. Int J Surg Case Rep.

[17] Wolosker N, de Campos JRM, Kauffman P (2022). Cohort study on 20 years' experience of bilateral video-assisted thoracic sympathectomy (VATS) for treatment of hyperhidrosis in 2431 patients. Sao Paulo Med J.

[18] Puri HV, Asaf BB, Bishnoi S, Pulle MV, Sharma S, Kumar A (2021). Thoracoscopic bilateral dorsal sympathectomy for primary palmo-axillary hyperhidrosis short- and mid-term results. J Minim Access Surg.

[19] Somma J, Couture ÉJ, Pelletier S (2021). Non-ventilated lung deflation during one-lung ventilation with a double-lumen endotracheal tube: a randomized-controlled trial of occluding the non-ventilated endobronchial lumen before pleural opening. Can J Anaesth.

[20] Lan L, Qiu Y, Zhang CZ, Ma TT, Cen YY (2020). Comparison of single-stage and two-stage bilateral video-assisted thoracic surgery. J Int Med Res.

[21] Chen J, Volpi S, Ali JM (2020). Comparison of post-operative pain and quality of life between uniportal subxiphoid and intercostal video-assisted thoracoscopic lobectomy. J Thorac Dis.

[22] Zhang Y, Wang Y, Lv C, Shu X, Wang J, Yang Q (2018). Clinical analysis of 56 cases of simultaneous bilateral video-assisted thoracoscopic surgery for bilateral synchronous multiple primary lung adenocarcinoma. J Thorac Dis.

[23] Cornwell LD, Echeverria AE, Samuelian J (2018). Video-assisted thoracoscopic lobectomy is associated with greater recurrence-free survival than stereotactic body radiotherapy for clinical stage I lung cancer. J Thorac Cardiovasc Surg.

[24] Chiu CH, Chao YK, Liu YH (2018). Subxiphoid approach for video-assisted thoracoscopic surgery: an update. J Thorac Dis.

[25] Bao F, Yu F, Wang R (2021). Electromagnetic bronchoscopy guided microwave ablation for early stage lung cancer presenting as ground glass nodule. Transl Lung Cancer Res.

